# Presence of microorganisms in children with pharyngotonsillitis and healthy controls: a prospective study in primary healthcare

**DOI:** 10.1007/s15010-021-01595-9

**Published:** 2021-03-08

**Authors:** Jon Pallon, Martin Sundqvist, Mattias Rööst, Patrik Danielsson, Thomas Neumark, Susann Skovbjerg, Jonas Svedin, Katarina Hedin

**Affiliations:** 1 Department of Clinical Sciences in Malmö, Family Medicine, Lund University, Malmö, Sweden; 2 Department of Research and Development, Region Kronoberg, Växjö, Sweden; 3 Clinical Research Centre, Institutionskansliet För Kliniska Vetenskaper, Box 50332, 202 13 Malmö, Sweden; 4 Department of Laboratory Medicine, Clinical Microbiology, Faculty of Medicine and Health, Örebro University, Örebro, Sweden; 5 Cityläkarna Primary Healthcare Centre, Region Kalmar County, Kalmar, Sweden; 6 Region Kalmar County, Kalmar, Sweden; 7 Department of Infectious Diseases, Institute of Biomedicine, Sahlgrenska Academy, University of Gothenburg, Gothenburg, Sweden; 8 Department of Clinical Microbiology, Sahlgrenska University Hospital, Region Västra Götaland, Gothenburg, Sweden; 9 Centre for Antibiotic Resistance Research (CARe), University of Gothenburg, Gothenburg, Sweden; 10 Anderslöv Primary Healthcare Centre, Region Skåne, Anderslöv, Sweden; 11 Futurum, Region Jönköping County, and Department of Health, Medicine and Caring Sciences, Linköping University, Linköping, Sweden

**Keywords:** Pharyngotonsillitis, Aetiology, Children, Primary healthcare, PCR, Prospective

## Abstract

**Purpose:**

Most studies on paediatric pharyngotonsillitis focus on group A streptococci. This study, however, analyses a broad spectrum of bacteria and viruses related to paediatric pharyngotonsillitis and evaluates their associated clinical symptoms and courses.

**Methods:**

This observational prospective study in primary healthcare includes 77 children aged < 15 with a sore throat and 34 asymptomatic children, all of whom were sampled from the tonsils with an E-swab^®^ for analysis with culture and PCR for 14 bacteria and 15 viruses. Patients were evaluated clinically, and their symptoms recorded in diaries for 10 days. Participants were followed up for 3 months by reviewing medical records.

**Results:**

A pathogen was detected in 86% of patients and in 71% of controls (*P* = 0.06). Bacteria were found in 69% of patients and 59% of controls (*P* = 0.3), and viruses in 36% and 26%, respectively (*P* = 0.3). Group A streptococci was the most common finding, with a prevalence of 49% and 32%, respectively (*P* = 0.1). Clinical signs were not useful for distinguishing pathogens. None of the controls and 16% of the patients reconsulted for a sore throat within 3 months.

**Conclusion:**

Bacteria were more common than viruses in both study groups. The high rate of pathogens in asymptomatic children interferes with diagnoses based on aetiology.

**Supplementary Information:**

The online version contains supplementary material available at 10.1007/s15010-021-01595-9.

## Introduction

Pharyngotonsillitis accounts for 6% of all primary healthcare visits by children [1] and leads to antibiotic prescriptions in 53–60% of the cases [1–4]. The most important pathogen is the bacterium *Streptococcus pyogenes* (group A streptococcus; GAS), which can cause both severe non-suppurative complications such as acute rheumatic fever and glomerulonephritis and immediate suppurative complications such as peritonsillar abscess, otitis media, and sinusitis. Non-suppurative complications are almost absent in high-income countries, and suppurative complications are too rare to justify antibiotic treatment. Current guidelines note that acute sore throat is a self-limiting infection that usually subsides within a week without antibiotic treatment, so the benefits of antibiotics must be weighed against adverse effects [5, 6].

Although GAS is the most common bacterial aetiology, it is only found in every third child with an acute sore throat and even less so in children younger than 5 years old [7]; that is, a majority of throat infections are caused by other pathogens, including respiratory viruses and other streptococcal species [8]. However, previous studies have often focused on a narrow spectrum of pathogens and relied on older methods such as culture and antigen detection [9, 10]. Moreover, GAS is also found in 12% of asymptomatic children [7], which poses problems diagnosing test-positive patients.

A few studies of unselected children with an acute sore throat in primary healthcare have investigated a broad range of respiratory pathogens using both culture and molecular methods [6]. In addition, there is a knowledge gap regarding the presentation and clinical course associated with these pathogens as well as their carriage rate in healthy children.

This study has three aims: (1) to estimate the prevalence of 29 respiratory pathogens in children with an acute sore throat and in healthy controls; (2) to relate signs, symptoms, and clinical course to aetiology; and (3) to measure the incidence of complications and return visits for a sore throat within 3 months after clinical examination.

## Materials and methods

### Design and setting

In this prospective inception cohort study, we recruited children with an acute sore throat in primary healthcare and studied their symptoms and clinical course in relation to detected pathogens. For comparison, we also included non-infected controls. Both groups were followed for 3 months regarding recurrence and complications. Four primary healthcare centres in three counties in southern Sweden participated. Inclusion was open between 12 September 2014 and 17 October 2017.

### Participants

Patients with suspected pharyngotonsillitis were initially identified by a triage nurse during a telephone assessment. During office hours for ordinary ambulatory care, these patients and their parents were recruited to participate by the authors and other physicians. These patients were eligible if they were 0–14 years old and had a sore throat lasting less than 7 days as a major complaint (or signs of pharyngotonsillitis on clinical examination in the youngest). Exclusion criteria were imminent complications associated with a sore throat (peritonsillitis, sinusitis, acute otitis media, or lymphadenitis colli), symptoms of obstructive airway disease, and difficulties understanding Swedish. Apart from study-related procedures, all patients received care-as-usual, including any required tests or prescriptions.

The control group was recruited from asymptomatic children aged 0–14 who belonged to the same primary healthcare centre and sought care for non-infectious conditions.

We set out for a consecutive sampling of all eligible patients, but as the researchers were not always in the office and the triage nurses at times forgot about the study, we ended up using convenience sampling.

### Data collection

After informed consent, the physician recorded background information on all participants. For patients, the physician also recorded signs and symptoms, working diagnosis, and decisions about antibiotics and ordered tests.

### Symptom diary

We asked the parents to keep a structured diary for 10 days and record symptoms (e.g., sore throat, stuffed up or runny nose, pain when swallowing, cough, hoarseness, diarrhoea, vomiting, and resting more than half the day), analgesics use, antibiotics use, and morning temperature. We also asked them to assess daily if their child was still unwell and if their children missed preschool or school due to their illness. After completion, they returned the diary by mail in a prepaid envelope. Two weeks after inclusion, we called each patient as a reminder.

### Microbiological sampling

Either the physician or trained staff at the primary healthcare centre collected a throat specimen from each participant by rolling a single nylon-flocked swab (E-Swab^®^, Copan Diagnostics Inc., Murrieta, CA) repeatedly against both tonsils. The swab was transferred to liquid Amies medium in the accompanying container and stored in a refrigerator for overnight transport. All samples were analysed the following day at the Department of Clinical Microbiology, Sahlgrenska University Hospital, Gothenburg, Sweden. To ensure analysis was performed the day following collection, we limited inclusion to Monday through Thursday between 8 a.m. and 4 p.m. The laboratory staff were blinded to clinical data and any point-of-care test results.

### Bacterial culture

A calibrated loop (10 μl) of diluted tonsillitis secretion was inoculated onto horse blood agar, *Streptococcus* agar, *Haemophilus* agar, and *Arcanobacterium haemolyticum* agar (all prepared in-house at Clinical Microbiology, Sahlgrenska University Hospital). The agar plates were incubated for 1 day at 34–36 °C in air with 5% CO_2_, and after inspection incubated for another day at 34–36 °C in air, or for the *Arcanobacterium* agar, in air with 5% CO_2_. Group A, B, C, and G streptococci, *Streptococcus pneumoniae*, *Haemophilus influenzae*, *Moraxella catarrhalis*, *Staphylococcus aureus*, and Gram-negative rods were enumerated and identified using standard bacteriological methods. *A. haemolyticum* was identified with a CAMP inhibition test.

### PCR detection of Fusobacterium necrophorum

Bacterial DNA was extracted and purified from 500 µl of diluted tonsillitis secretion using Amplicor Respiratory Specimen Preparation kits (Roche Diagnostics, Mannheim, Germany). *F. necrophorum* ssp. *funduliforme* was detected with a real-time PCR using previously published primers for the *rpo* gene (partial) [11], and SYBR green for detection of the amplified PCR product. The PCR conditions were as follows: initial denaturation at 95 °C for 2 min, followed by 40 cycles, each cycle consisting of 95 °C for 15 s, 60 °C for 15 s, and 72 °C for 20 s, all performed in a Rotor-Gene Q (Qiagen, Sollentuna, Sweden). After a pre-incubation step at 75 °C for 90 s, a melting curve analysis was performed from 75 to 95 °C, rising by one degree each step, to confirm the correct *F. necrophorum rpo* gene amplification.

### PCR detection of viral and other bacterial pathogens

Nucleic acids from 200 μl of the tonsillitis secretion were extracted with a MagNA Pure LC instrument (Roche Diagnostics, Mannheim, Germany) using Total Nucleic Acid Isolation kits (Roche Diagnostic). Next, a multiplex real-time PCR was performed to detect 15 respiratory tract viruses (adenovirus, bocavirus, coronavirus 229E, OC43, NL63 and HKU-1, enterovirus, influenza A and B virus, metapneumovirus, parainfluenza virus 1–3, rhinovirus and respiratory syncytial virus, RSV) and five bacteria (*S. pneumoniae*, *H. influenzae*, *Bordetella pertussis*, *Chlamydophila pneumoniae,* and *Mycoplasma pneumoniae*) [12].

### Follow-up

Three months after inclusion, we reviewed the medical records of all patients and controls regarding return visits for a sore throat during the period and for a complication (peritonsillitis, sinusitis, acute otitis media, lymphadenitis colli, glomerulonephritis, or rheumatic fever) within 30 days of inclusion. We had access to relevant data from primary healthcare and hospitals at all study sites.

### Statistical analyses

Based on earlier reports [7, 9], we estimated that 100 patients and 100 controls would be sufficient to describe the epidemiologic situation and to reveal possible differences in aetiological prevalence between groups, primarily regarding GAS.

Data were analysed with SPSS 23.0 (IBM, Armonk, NY, USA). Continuous variables with non-normal distribution or with small sample sizes were reported as median (interquartile range, IQR). For comparison of three or more groups of variables not normally distributed, we used Kruskal–Wallis *H* test, reported with the *H* statistic, degrees of freedom and *P* value. For comparison of categorical data, we used either Pearson *χ*^2^ or Fisher’s exact test for independent groups, and McNemar’s test for paired data.

Before analysis, the participants were grouped by age: < 1 year (before preschool), 1–5 years (preschool), and 6–14 years (school). The microorganisms were also grouped, partly because of small numbers and partly to reflect clinical usefulness: “GAS” (corresponding to a positive culture or a rapid antigen detection test), “any bacteria” (positive in culture and/or PCR), “only viruses” (no benefit from antibiotics), and “no detected pathogen”. We chose to use Centor score (one point each for fever, absence of cough, tonsillar coating, and tender cervical lymph glands) [13] rather than McIsaac score (age-adjusted Centor score) [14] to describe the summarized clinical features, because Centor score mirrors Swedish guidelines [15] and the two scoring systems are similar in the age group 3–14 years.

Aetiological predictive value, introduced by Gunnarsson and Lanke [16], is a statistical method that accounts for asymptomatic carriage when interpreting an aetiological test. As microbial carriage is also seen in symptomatic people, a positive finding could mean either infection or carriage. To correctly assess the test outcome, the level of uncertainty must first be quantified. Positive and negative predictive values with 95% confidence intervals can be calculated with known data for the prevalence of the pathogen (in our case GAS) for both patients and healthy subjects as well as the sensitivity of the test. It is also necessary to estimate “theta”—i.e., the ratio of GAS carriage in healthy individuals and in patients with a sore throat caused by a virus. Based on Gunnarsson and Lanke, we assumed a 90% sensitivity of throat culture to detect GAS and a theta of 0.9.

## Results

### Characteristics

The study included 79 patients and 34 controls. Two patients were later excluded from analysis due to withdrawn consent or symptoms lasting more than 7 days. Patients and controls were included in parallel, and most patients (63 of 77, 82%) and controls (28 of 34, 82%) were recruited during cold months (October–April). The age distribution was similar in both groups, with a median value of 7.8 years in patients (IQR 4.6–11) and 7.7 years in controls (IQR 4.2–10). Among the patients, 71 of 77 (92%) were aged 3 or older. The median number of days with symptoms before consultation was 3 (IQR 2–5). Other background characteristics of the study population are presented in Table 1.

**Table 1:** Characteristics of the study population

	Number (%)		
	Patients (*n* = 77)	Controls (*n* = 34)	*χ*^2^ (Fisher)
			*P* value
Age 0	2 (3)	0	1 (Fisher)
Age 1–5	27 (35)	11 (32)	0.8
Age 6–14	48 (62)	23 (68)	0.8
Female	52 (68)	16 (47)	0.04
Smoker in household	11 (14)	5 (15)	1
A history of recurring sore throat	25 (32)	3 (9)	0.008
Previous tonsillectomy	5 (6)	2 (6)	1 (Fisher)
Antibiotic treatment in the last month	10 (13)	0	0.03 (Fisher)
Prone to infections (parents’ view)	16 (21)	1 (3)	0.02
Sore throat in family member in the last month	49 (64)	13 (38)	0.01

### Detected pathogens

#### Prevalence

In 66 of 77 patients (86%) and 24 of 34 controls (71%), we detected at least one of the 29 targeted pathogens (*P* = 0.06). Bacteria were found in 69% of the patients and 59% of the controls (*P* = 0.3), and viruses in 36% and 26%, respectively (*P* = 0.3). That is, bacteria were more common than viruses among both patients (*P* = 0.001) and controls (*P* = 0.02). Thirteen of the pathogens were never detected in the patients, and 17 were never detected in the controls.

GAS was the most prevalent pathogen in patients, making up a majority of bacterial findings, followed by *H. influenzae*, *S. aureus*, influenza B virus, and rhinovirus. In controls, GAS was also the most prevalent pathogen, followed by rhinovirus (Tables 2 and 3). We detected two or three concomitant pathogens in 23 patients (30%), 15 of which were a combination of bacteria and viruses. The most common combination was GAS and influenza B virus (*n* = 4). Nine (26%) of the controls had two or three concomitant pathogens. GAS was mostly detected as a sole pathogen (in 71% of patients and 55% of controls with GAS, respectively).

**Table 2:** Bacteria and viruses detected by culture or PCR in children with a sore throat and in controls

	Number of patients (%)		
	Patients (*n* = 77)	Controls (*n* = 34)	Fisher or *χ*^2^
			*P*
Bacteria			
Group A streptococci	38 (49)	11 (32)	0.1^e^
Group C streptococci	1 (1)	3 (9)	0.08
Group G streptococci	–	1 (3)	0.3
*Haemophilus influenzae*	9 (12)^a^	2 (6)^a^	0.5
*Fusobacterium necrophorum*	1 (1)	1 (3)	0.5
*Mycoplasma pneumoniae*	–	1 (3)	0.3
*Staphylococcus aureus*	7 (9)	3 (9)	1
Gram-negative rods	5 (6)^b^	3 (9)^c^	0.7
Any bacteria	53 (69)	20 (59)	0.3^e^
Viruses			
Adenovirus	4 (5)	–	0.3
Bocavirus	–	2 (6)	0.1
Coronavirus NL63	1 (1)	1 (3)	0.5
Coronavirus OC43	1 (1)	–	1
Enterovirus	4 (5)^d^	1 (3)	1
Influenza A virus	2 (3)	–	1
Influenza B virus	6 (8)	–	0.2
Metapneumovirus	3 (4)	–	0.6
Parainfluenzavirus 1	1 (1)	–	1
Rhinovirus	7 (9)	7 (21)	0.1^e^
Respiratory syncytial virus	2 (3)	–	1
Any virus	28 (36)	9 (26)	0.3^e^

The following bacteria and viruses were not detected: *Arcanobacterium haemolyticum*, *Bordetella pertussis*, *Chlamydophila pneumoniae*, group B streptococci, *Moraxella catarrhalis*, *Streptococcus pneumoniae*, Coronavirus 229E and HKU-1, Parainfluenzavirus 2 and 3

^a^*Haemophilus influenzae* was detected in patients both as the sole finding (*n* = 2), and concomitant with group A streptococci (*n* = 2), *S. aureus* (*n* = 3), and viruses (*n* = 3). Among controls, it was detected together with a virus (*n* = 1) and *M. pneumoniae* (*n* = 1)

^b^*Enterobacter cloacae* (*n* = 1), *Klebsiella pneumoniae* (*n* = 4)

^c^*Pseudomonas* spp. (*n* = 3)

^d^In one patient, the analysis could not differentiate between enterovirus and rhinovirus

^e^*χ*^2^ test was used

**Table 3:** Aetiology vs. age in children < 15 years with a sore throat

	Aetiology, *n* (%)								
	All ages^a^			Age 1–5			Age 6–14		
	Patients (*n* = 77)	Controls (*n* = 34)	*P* value	Patients (*n* = 27)	Controls (*n* = 11)	*P* value	Patients (*n* = 48)	Controls (*n* = 23)	*P* value
Any pathogen	66 (86)	24 (71)	0.06	24 (89)	9 (82)	0.6^b^	40 (83)	15 (65)	0.09
Any bacteria	53 (69)	20 (59)	0.3	18 (67)	6 (55)	0.7^b^	33 (69)	14 (61)	0.5
GAS	38 (49)	11 (32)	0.1	13 (48)	2 (18)	0.1^b^	25 (52)	9 (39)	0.3
Only viruses	13 (17)	4 (12)	0.5	6 (22)	3 (27)	1^b^	7 (15)	1 (4)	0.3^b^

*P* values are for Pearson *χ*^2^ test

*GAS* group A streptococci

^a^“All ages” also includes the two patients aged < 1 year

^b^Fisher’s exact test

#### Aetiology and age

No pathogen was detected in the two patients who were under 1 year old. In the two older age groups, the distribution of pathogens in each group mirrored the overall pattern, and we found no differences between patients and controls that were statically significant (i.e., *P* < 0.05). The relationship between age group and microbial findings is presented in Table 3.

#### Aetiological predictive value for group A streptococci

With a prevalence of 49% for patients and 32% for controls, the positive aetiological predictive value for GAS was 54% (95% CI 0–92%). Restricting the calculation to patients with a Centor score of 3–4, the corresponding value was 67% (95% CI 0–97%).

### Clinical symptoms and management

#### Symptoms and aetiology

The median number of days with a sore throat before consultation was similar between the mutually exclusive groups “any bacteria”, “only viruses”, and “no pathogen” (*H* = 2.5, 2 *d.f.*, *P* = 0.3) (Table 4). Swollen tonsils were found in 47% of patients with GAS and 31% of patients with only viruses (*P* = 0.3), and had a positive predictive value of 67% for GAS (95% CI 51–80%). Tender cervical lymph glands were common both in patients with GAS and in patients with “no pathogen” and had a positive predictive value of 53% for GAS (95% CI 41–64%). Coryza was more common in patients with only viruses than in patients with GAS (*P* = 0.04), but it had a low positive predictive value for viruses (24%; 95% CI 16–35%). A cough was present in 46% of patients with only viruses and 24% of those with GAS (*P* = 0.2). A lack of a cough had a positive predictive value for GAS of 55% (95% CI 47–62%).

**Table 4:** Clinical signs vs. pathogen findings in children < 15 years with a sore throat, *n* (%)

	All patients (*n* = 77)	Any bacteria (*n* = 53)	GAS (*n* = 38)	Only viruses (*n* = 13)	No pathogen (*n* = 11)
Days with a sore throat prior to visit, median (IQR)	3 (2–5)	3 (2.3–4.8)	3 (2–4.5)	3 (2–5.5)	2 (1–4)
Cough	24 (31)	16 (30)	9 (24)	6 (46)	2 (18)
Coryza	33 (43)	20 (38)	11 (29)	8 (62)	5 (45)
Tender cervical lymph glands	35 (45)	24 (45)	19 (50)	4 (31)	7 (64)
Tonsillar coating	19 (25)	13 (25)	9 (24)	3 (23)	3 (27)
Tonsillar erythema	54 (70)	38 (72)	29 (76)	8 (62)	8 (73)
Swollen tonsils	27 (35)	21 (40)	18 (47)	4 (31)	2 (18)
Petechiae	5 (6)	4 (8)	3 (8)	1 (8)	–
Raspberry tongue	1 (1)	1 (2)	1 (3)	–	–
Scarlatine rash	1 (1)	1 (2)	1 (3)	–	–
Impetigo	–	–	–	–	–
Temperature ≥ 38.5 °C	7 (9)	3 (6)	3 (8)	3 (23)	1 (9)
Centor score					
0	6 (8)	3 (6)	2 (5)	3 (23)	–
1	19 (25)	14 (26)	6 (16)	2 (15)	3 (27)
2	22 (29)	16 (30)	13 (34)	2 (15)	2 (18)
3	30 (39)	20 (38)	17 (45)	4 (31)	6 (55)
4	–	–	–	–	–

*GAS* group A streptococci

#### Centor scores

In total, 47 of 77 patients (61%) had a Centor score of 0–2, and 30 patients (39%) had a score of 3 (Table 4). As there were few patients with fever at consultation (*n* = 9), no patient had a score of 4. A Centor score of 3 was seen in 45% of patients with GAS and in 31% of patients with only viruses (*P* = 0.5). The positive predictive value of a Centor score of 3–4 for GAS was 57% (95% CI 43–70%) and the negative predictive value was 55% (95% CI 46–64%).

### Clinical course

#### Symptom diaries

We received complete diaries from 55 of 77 patients (71%). The response rate differed slightly between the groups: 74% for “any bacteria”, 77% for “only viruses”, and 55% for “no pathogen” (*P* = 0.4, Fisher). Most of these patients (52 of 55) reported a resolution of their sore throat within 10 days, although five experienced recurrent symptoms.

**Fig. 1:**
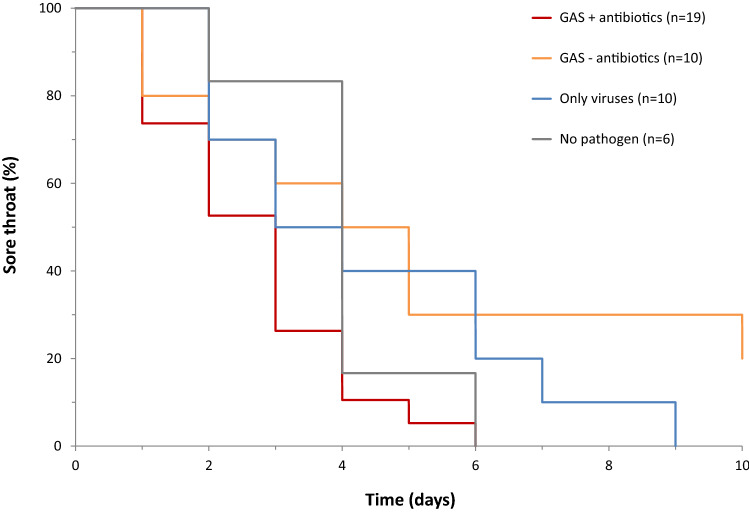
Duration of a sore throat after a visit to a physician, as reported in symptom diaries of 55 children aged 0–14. *GAS* group A streptococci, with and without antibiotic treatment

The median duration of a sore throat after consultation differed between groups, with the fastest resolution in GAS patients treated with antibiotics (median 3 days; IQR 1.5–3.5) and the slowest resolution in GAS patients not treated with antibiotics (median 4.5 days; IQR 2.3–8.8). The difference, however, was not statistically significant (*H* = 6.2, 3 *d.f.*, *P* = 0.1). The gradual resolution of a sore throat is illustrated in Fig. 1.

Self-reported prevalence of a sore throat, fever, and absence from preschool or school at days 3 and 7 in the different groups are presented in Supplementary Table 1.

#### Three-month follow-up

All 77 patients and 34 controls were followed up after 3 months. Twelve patients (16%) had made return visits for a sore throat after a median of 25 days (IQR 18–53). None of the patients and controls had a complication and none were hospitalized.

Four of the twelve patients reported worsened or non-resolving symptoms, and they all had non-treated GAS at inclusion. The other eight patients reported a new episode, and five of these had GAS at inclusion, three of whom received antibiotics. None of the controls consulted for a sore throat during the follow-up.

## Discussion

In this prospective observational study on pharyngotonsillitis in children presenting to primary healthcare, we found a high prevalence of bacteria and viruses in both patients (86%) and controls (71%). Bacteria were more common than viruses in both groups, and GAS was the most common pathogen. The observed differences in signs and symptoms between bacteria and viruses were not specific enough to be clinically useful. The fastest resolution of symptoms was seen in GAS patients treated with antibiotics. After 3 months, 16% of the patients had made return visits for a sore throat, but without a clear association to detected pathogens.

### Strengths and weaknesses

To our knowledge, this is the first study on self-referred and unselected children with pharyngotonsillitis in primary healthcare that takes advantage of PCR to screen for a broad range of pathogens in both patients and controls and associates those findings with clinical symptoms and the course of the infection. Despite the lack of specific demographic data, we believe that this multicentre study is representative of children presenting to primary care with an acute sore throat. The data were collected in both urban and rural areas over three seasons, and the findings are reported by age strata to further increase their usefulness. Whether our findings can be replicated elsewhere depends on the epidemiological situation in those locations.

The low number of participants, especially controls, was less than we aimed for, and this could have introduced type II errors. Based on previous data, we expected to recruit a sufficient number of participants in one season, but failed to do so, mainly because the clinics were unable to provide enough resources. We also learned that children visiting for non-infectious causes are scarce, and they may not want to participate in a study while suffering from a sprained ankle or upset stomach. Aware of this limitation, we urge the reader to consider this an exploratory study.

Some methodological limitations need to be discussed. First, as we did not ask about fever previous to the visit, we might have missed important information, especially since the proportion of children with fever at the clinic was lower than expected (possibly explained by uncalibrated thermometers, use of antipyretics, or many visits in the morning). Second, although a throat swab may be more convenient for children than a nasopharyngeal swab and better reflect the pathogens of a pharyngeal infection, this technique could be an inferior way to detect viruses and result in false negatives [17]. Regardless of technique, because aetiological tests only test for the specified microorganisms, we probably missed other pathogens. By adding biomarker tests, we might have been able to classify the infection as viral or bacterial [18]. Third, as many diaries were never returned, we should have used other ways to obtain the information and help the parents, for example, by offering web-based forms.

### Interpretation

Group A streptococcus (GAS) was the most prevalent pathogen in both patients and controls, a finding in line with the previous reports [7]. However, the high carriage rate made us wonder if there had been an outbreak of GAS during the study period; analysis of the temporal variations revealed no such fluctuations (data not shown). Normally, GAS in children under 5 years old is less prevalent than in older children, but our study could only confirm this in the controls, not in the patients. Group C or G streptococci were only found in one patient but in four of 34 controls, a finding congruent with a large observational study that suggests both an increasing incidence with age and a likely carriage state in children [19].

*Haemophilus influenzae*, *S. aureus*, and *K. pneumoniae* were found in a quarter of patients, as well as in controls. Although these bacteria can be associated with disease in children, they are more likely to represent a colonization [20]. *M. catarrhalis*, another common bacterium in the nasopharyngeal microbiome of children, was never detected [5].

The anaerobic bacterium *Fusobacterium necrophorum* has been suggested as a possible pathogen in adolescents with pharyngitis [21–23]. We detected *F. necrophorum* in only one patient, aged 14 and with a concomitant finding of influenza B virus, and in one control, aged 3. These findings are in line with a previous report of a 2% prevalence in children under 15 years old [23].

The prevalence of viruses in our study was much lower than the prevalence of viruses from a previous study using PCR [18]. This unexpected finding could be the result of the sampling errors described above, age distribution differences between our study and the previous studies, and epidemiologic differences between our settings and the previous study’s settings. Among children, viruses become less prevalent with age [18], and two-thirds of our patients were 6–14 years old.

Rhinovirus was the most prevalent virus in both patients and controls, which is congruent with studies using PCR [18, 24], while adenovirus, the most prevalent virus in older studies [9, 10, 25], was less common. In our study, parainfluenzavirus, metapneumovirus, and RSV were only found in patients, which supports the findings of a study on young children with acute respiratory infection [24].

The high rate of bacteria and viruses in asymptomatic children makes it difficult to interpret a positive finding in patients, as there is good reason to assume that they have similar carriage rates [16]. This is especially true for GAS [7], rhinoviruses [24, 26], and adenoviruses [18]. The fact that most findings in our study were single pathogens does not contradict the idea of a simultaneous carriage and infection, as we had no test for aetiological causality and no estimate of false-negative findings. Both rapid antigen tests and the Centor criteria are used to detect GAS, not to distinguish between infection and colonization.

While detection of microorganisms is insufficient for determining causality, measuring the host response may get us closer. Repeated testing for streptococcal antibodies could retrospectively determine a likely infection with GAS [27], but this will not help the clinician at the time of visit. C-reactive protein (CRP) and procalcitonin are biomarkers that have been suggested to distinguish bacterial from viral infections, but their usefulness lies in repeated measures in hospitalized patients, and have not been proven useful in diagnosing pharyngitis in adults [6]. Myxovirus resistance protein A (MxA) is a marker for viral infections, and a recent study found a clear association between elevated MxA levels and detection of viruses in children with febrile pharyngitis [18]. However, the differential diagnostic value for bacterial infection was poor, as an elevated MxA does not exclude a concomitant finding of GAS. Combining MxA with CRP could be a better approach, but this needs more evaluation [18]. Transcriptional profiling is another promising technique to differentiate viral detection from an active viral infection [26].

Rather than relying on biomarkers, the statistical method etiologic predictive value (EPV) considers asymptomatic carriage when interpreting an aetiological finding in patients [16]. Although this approach does not answer the question of causality, it does provide an important indication of the uncertainty. In our study, we found that the EPV of a GAS-positive culture was only 54%, no more than flipping a coin, and with an incredibly wide confidence interval due to the high carriage rate. Incidentally, a recent meta-analysis found that only 56% of children with GAS-positive had a serologically confirmed infection [28].

The large diagnostic uncertainty must also be weighed against the small clinical benefits of antibiotic treatment, the low risk of complications in untreated patients, and the adverse effects of antibiotics. Except for patients with severe symptoms, no prescription or a back-up prescription could therefore be a better approach, which is in line with current guidelines [5, 6].

Our study adds to previous knowledge [9, 18, 29, 30] by noting that the clinical presentation for viruses and bacteria was very similar. Viral features like cough and coryza were less common in patients with GAS, but as GAS was highly prevalent, the positive predictive values for viruses for these symptoms were still low. No single symptom was specific enough for GAS or viruses to change the post-test probability to > 85%, a level of reasonable certainty that approaches the performance of a rapid antigen detection test [30]. Although pointing to difficulties in aetiological diagnosis in children with pharyngotonsillitis, we do not consider the results of this small descriptive study robust enough to change clinical guidelines.

## Conclusion

With a high carriage rate of both viruses and bacteria among controls, it is likely that symptomatic patients also harbour these microorganisms alongside their active infection. Together with the low predictive values of signs and symptoms, this makes causal aetiological diagnosis in children with pharyngotonsillitis very challenging, even where rapid antigen detection tests are available. The development of a fast, specific, and cheap point-of-care marker for active infection would be of great value.

## Supplementary Information

Below is the link to the electronic supplementary material.

Supplementary file1 (DOCX 13 KB)

## Data Availability

The datasets generated and analysed during the current study are not publicly available due to Swedish legislation (the Personal Data Act), but are available from the corresponding author on reasonable request.

## Abbreviations

GAS: Group A streptococci
PCR: Polymerase chain reaction
