# Supplementary material for: Presence of microorganisms in children with pharyngotonsillitis and healthy controls: a prospective study in primary healthcare
Source: Infection. 2021 Mar 8;49(4):715–24. doi: 10.1007/s15010-021-01595-9 (PMC7938884; doi:10.1007/s15010-021-01595-9)
Supplement: Supplementary file 1 — Supplementary file1 (DOCX 13 KB) [file 15010_2021_1595_MOESM1_ESM.docx]

| **SUPPLEMENTARY TABLE 1. Self-reported symptoms in diaries of children with a sore throat, n (%)** | | | | |
| --- | --- | --- | --- | --- |
|  | **GAS + antibiotics**  **(n=19)** | **GAS – antibiotics**  **(n=10)** | **Only viruses**  **(n=10)** | **No pathogen**  **(n=6)** |
| Sore throat |  |  |  |  |
| Day 3 | 5 (26) | 6 (60) | 5 (50) | 5 (83) |
| Day 7 | 0 | 3 (30) | 1 (10) | 0 |
| Fever ≥ 38.5 °C |  |  |  |  |
| Day 3 | 0 | 0 | 2 (20) | 1 (17) |
| Day 7 | 0 | 0 | 0 | 1 (17) |
| Absence from preschool/school |  |  |  |  |
| Day 3 | 4 (21) | 2 (20) | 6 (60) | 2 (33) |
| Day 7 | 1 (5) | 2 (20) | 0 | 2 (33) |
| GAS = group A streptococci | | | | |
